# 9-(2,4-Di­fluoro­phen­yl)-3,3,6,6-tetra­methyl-3,4,5,6,7,9-hexa­hydro-2*H*-xanthene-1,8-dione

**DOI:** 10.1107/S1600536814002761

**Published:** 2014-02-12

**Authors:** S. Rizwana Begum, R. Hema, G. Sumathi, R. Valliappan, N. Srinivasan

**Affiliations:** a Department of Physics, Seethalakshmi Ramaswami College (Autonomous), Tiruchirappalli 620 002, India; b Department of Physics, K. Ramakrishnan College of Engineering, Samayapuram, Tiruchirappalli 621 112, India; c Department of Chemistry, Sri Krishna Engineering College, Panapakkam, Thambaram 601 301, India; d Department of Chemistry, Chemistry Wing DDE, Annamalai University, Annamalainagar 608 002, India; e Department of Chemistry, S.K.P. Engineering College, Thiruvanamalai 606 611, India

## Abstract

In the title compound, C_23_H_24_F_2_O_3_, the central pyran ring has a flat-boat conformation, whereas the two fused cyclo­hexenone rings adopt envelope conformations, with the C atom bearing the dimethyl substituent being the flap atom in each case. The pyran ring mean plane and the di­fluoro­phenyl ring are almost normal to each other, making a dihedral angle of 87.55 (4)°. In the crystal, mol­ecules are linked by pairs of C—H⋯O hydrogen bonds, forming inversion dimers with an *R*_2_^2^(8) ring motif. The F atom at position 2 on the di­fluoro­phenyl ring is disordered over the 2- and 6-positions, and has a refined occupancy ratio of 0.932 (3):0.068 (3).

## Related literature   

For the synthesis of xanthenes, see: Vanag & Stankevich (1960 ▶); Hilderbrand & Weissleder (2007 ▶). For their pharmaceutical properties, see: Jonathan *et al.* (1988 ▶); Lambert *et al.* (1997 ▶); Poupelin *et al.* (1978 ▶); Hideo (1981 ▶); Selvanayagam *et al.* (1996 ▶). For related structures, see: Sughanya & Sureshbabu (2012 ▶); Sureshbabu & Sughanya (2013 ▶). For ring conformation analysis, see: Cremer & Pople (1975 ▶). For hydrogen-bonding graph-set motifs, see: Bernstein *et al.* (1995 ▶).
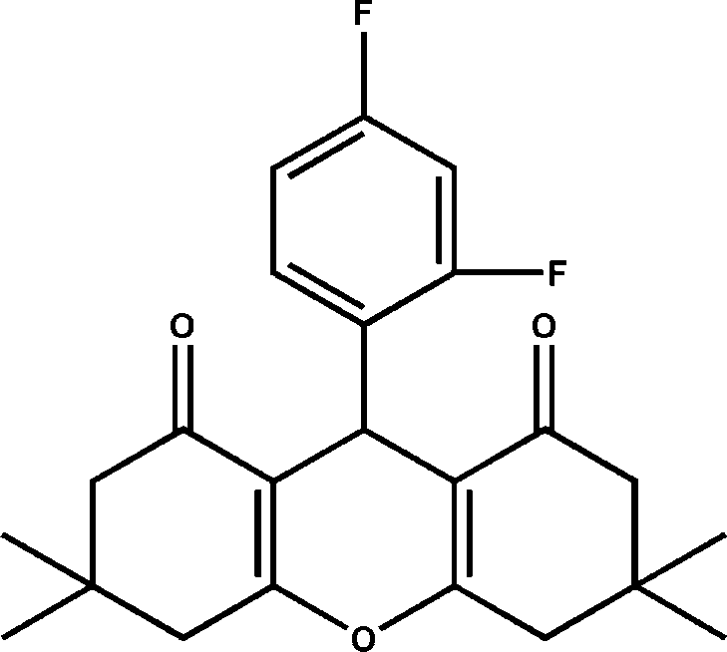


## Experimental   

### 

#### Crystal data  

C_23_H_24_F_2_O_3_
*M* *_r_* = 386.42
Triclinic, 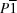

*a* = 9.6810 (4) Å
*b* = 10.4290 (4) Å
*c* = 11.8840 (5) Å
α = 69.288 (2)°
β = 74.895 (2)°
γ = 63.406 (2)°
*V* = 996.03 (7) Å^3^
*Z* = 2
Mo *K*α radiation
μ = 0.10 mm^−1^
*T* = 293 K
0.30 × 0.20 × 0.20 mm

#### Data collection  

Bruker Kappa APEXII CCD diffractometer
Absorption correction: multi-scan (*SADABS*; Bruker, 2004 ▶) *T*_min_ = 0.972, *T*_max_ = 0.981
23107 measured reflections
6738 independent reflections
4248 reflections with *I* > 2σ(*I*)
*R*_int_ = 0.026

#### Refinement  

*R*[*F* ^2^ > 2σ(*F* ^2^)] = 0.048
*wR*(*F* ^2^) = 0.147
*S* = 1.03
6738 reflections
267 parameters
H-atom parameters constrained
Δρ_max_ = 0.24 e Å^−3^
Δρ_min_ = −0.18 e Å^−3^

### 

Data collection: *APEX2* (Bruker, 2004 ▶); cell refinement: *SAINT* (Bruker, 2004 ▶); data reduction: *SAINT* and *XPREP* (Bruker, 2004 ▶); program(s) used to solve structure: *SIR92* (Altomare *et al.*, 1993 ▶); program(s) used to refine structure: *SHELXL2013* (Sheldrick, 2008 ▶); molecular graphics: *ORTEP-3 for Windows* (Farrugia, 2012 ▶); software used to prepare material for publication: *PLATON* (Spek, 2009 ▶).

## Supplementary Material

Crystal structure: contains datablock(s) I, global. DOI: 10.1107/S1600536814002761/su2696sup1.cif

Structure factors: contains datablock(s) I. DOI: 10.1107/S1600536814002761/su2696Isup2.hkl

Supporting information file. DOI: 10.1107/S1600536814002761/su2696Isup3.cml

CCDC reference:

Additional supporting information:  crystallographic information; 3D view; checkCIF report

## Figures and Tables

**Table 1:** Hydrogen-bond geometry (Å, °)

*D*—H⋯*A*	*D*—H	H⋯*A*	*D*⋯*A*	*D*—H⋯*A*
C20—H20⋯O1^i^	0.93	2.38	3.3075 (15)	177

Symmetry code: (i) 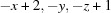.

**Fig. 1.**
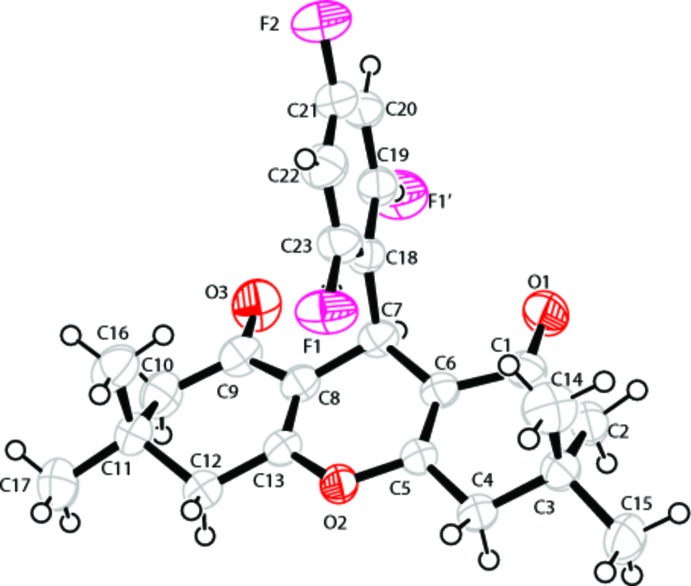
The molecular structure of the title compound, with atom labelling. Displacement ellipsoids are drawn at the 50% probability level.

**Fig. 2.**
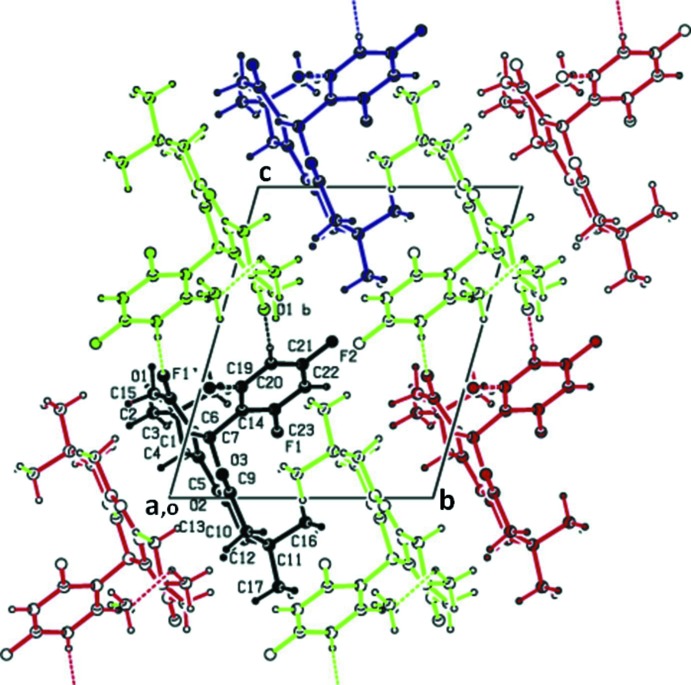
The crystal packing of the title compound viewed along the a axis. The C-H···O hydrogen bonds are shown as dashed lines - see Table 1 for details.

## supplementary crystallographic information

### 1. Comment

Xanthene is the parent compound of a number of naturally occurring substances and some synthetic dyes. Xanthene derivatives are used as dyes (Hilderbrand & Weissleder, 2007) and are used in medicine as they possess biological properties like antibacterial, antiviral and anti-inflammatory activities (Jonathan *et al.*, 1988). Ehretianone, a quinonoid xanthene was reported to possess anti-snake venom activity (Selvanayagam *et al.*, 1996; Lambert *et al.*, 1997; Poupelin *et al.*, 1978; Hideo, 1981). In view of the importance of the Xanthene derivatives we have synthesized the title compound and report herein on its crystal structure.

In the title molecule, Fig. 1, the pyran ring (O2/C5-C8/C13) has a flat boat conformation [Q = 0.1721 (13) Å, θ = 75.4 (4)° and φ = 186.9 (5)°] with a deviation for atoms O2 [0.1999 (12) Å] and C7 [0.0898 (9) Å] from the mean plane of the other four atoms. The two fused cyclohexenone rings adopt envelope conformations with puckering parameter (Cremer & Pople, 1975) Q = 0.4417 (15) Å, θ = 55.22 (19)° and φ = 116.4 (2)° for ring (C1-C6) and Q = 0.4715 (16) Å, θ = 59.08 (19)° and φ = 179.5 (2)° for ring (C8-C13). Atoms C3 and C11 are the flap atoms being situated out of the mean plane of the respective ring by 0.3118 (9) and 0.3324 (10) Å. The dihedral angle between the mean plane of the central pyran ring (O2/C5-C8/C13) and the difluorophenyl ring (C18-C23) is 87.55 (4)°.

In the crystal, molecules are linked by a pair of C-H···O hydrogen bonds (Fig. 2 and Table 1) forming inversion dimers with an *R*^2^_2_(8) ring motif (Bernstein *et al.*, 1995).

### 2. Experimental

5,5-dimethylcyclohexane-1,3-dione (1.15 g, 16 mmol) was treated with 2,4-difluorobenzaldehyde (0.6 g, 8 mmol) in ethanol (10 ml). The reaction mixture was heated for 5 min. After cooling to room temperature, a solid started to separate out. This solid was filtered, dried and then recrystallized from ethanol to yield colourless block-like crystals of the title compound [Yield 0.95 g; 80%].

### 3. Refinement

Atom F1 on the difluorophenyl ring is slightly disordered, being attached to atoms C23 and C19 with a refined occupancy ratio of 0.932 (3):0.068 (3), for atoms F1:F1' and H19:H23. The H atoms were included in calculated positions and treated as riding atoms: C-H = 0.93, 0.98, 0.97 and 0.96 Å for CH(aromatic), CH, CH_2_ and CH_3_ H atoms, respectively, with *U*_iso_(H) = 1.5*U*_eq_(C-methyl) and = 1.2*U*_eq_(C) for other H atoms.

### Crystal data

**Table:**

C_23_H_24_F_2_O_3_	*Z* = 2
*M**_r_* = 386.42	*F*(000) = 408
Triclinic, *P*1	*D*_x_ = 1.288 Mg m^−^^3^
*a* = 9.6810 (4) Å	Mo *K*α radiation, λ = 0.71073 Å
*b* = 10.4290 (4) Å	Cell parameters from 8029 reflections
*c* = 11.8840 (5) Å	θ = 2.8–30.5°
α = 69.288 (2)°	µ = 0.10 mm^−^^1^
β = 74.895 (2)°	*T* = 293 K
γ = 63.406 (2)°	Block, colourless
*V* = 996.03 (7) Å^3^	0.30 × 0.20 × 0.20 mm

### Data collection

**Table:**

Bruker Kappa APEXII CCD diffractometer	6738 independent reflections
Radiation source: fine-focus sealed tube	4248 reflections with *I* > 2σ(*I*)
Graphite monochromator	*R*_int_ = 0.026
ω and φ scan	θ_max_ = 31.9°, θ_min_ = 1.9°
Absorption correction: multi-scan (*SADABS*; Bruker, 2004)	*h* = −9→14
*T*_min_ = 0.972, *T*_max_ = 0.981	*k* = −10→15
23107 measured reflections	*l* = −17→17

### Refinement

**Table:**

Refinement on *F*^2^	0 restraints
Least-squares matrix: full	Hydrogen site location: inferred from neighbouring sites
*R*[*F*^2^ > 2σ(*F*^2^)] = 0.048	H-atom parameters constrained
*wR*(*F*^2^) = 0.147	*w* = 1/[σ^2^(*F*_o_^2^) + (0.0674*P*)^2^ + 0.1104*P*] where *P* = (*F*_o_^2^ + 2*F*_c_^2^)/3
*S* = 1.03	(Δ/σ)_max_ < 0.001
6738 reflections	Δρ_max_ = 0.24 e Å^−^^3^
267 parameters	Δρ_min_ = −0.18 e Å^−^^3^

### Special details

**Table:**

Geometry. All esds (except the esd in the dihedral angle between two l.s. planes) are estimated using the full covariance matrix. The cell esds are taken into account individually in the estimation of esds in distances, angles and torsion angles; correlations between esds in cell parameters are only used when they are defined by crystal symmetry. An approximate (isotropic) treatment of cell esds is used for estimating esds involving l.s. planes.

### Fractional atomic coordinates and isotropic or equivalent isotropic displacement parameters (Å^2^)

**Table:**

	*x*	*y*	*z*	*U*_iso_*/*U*_eq_	Occ. (<1)
F1	0.58177 (9)	0.33825 (10)	0.21213 (9)	0.0634 (3)	0.932 (3)
F1'	1.0560 (15)	0.0461 (17)	0.3554 (14)	0.078 (5)	0.068 (3)
F2	0.72300 (13)	0.44738 (12)	0.50286 (9)	0.0828 (3)	
O1	0.84119 (12)	−0.15460 (12)	0.39286 (9)	0.0627 (3)	
O2	0.65242 (9)	0.17594 (9)	0.02533 (7)	0.0434 (2)	
O3	1.11867 (11)	0.17515 (13)	0.07618 (10)	0.0686 (3)	
C1	0.74840 (14)	−0.10446 (13)	0.32037 (11)	0.0420 (3)	
C2	0.63396 (15)	−0.17246 (15)	0.33580 (11)	0.0473 (3)	
H2A	0.6041	−0.2087	0.4218	0.057*	
H2B	0.6856	−0.2578	0.3029	0.057*	
C3	0.48605 (14)	−0.06656 (14)	0.27502 (11)	0.0435 (3)	
C4	0.53389 (14)	0.00773 (14)	0.14367 (11)	0.0430 (3)	
H4A	0.5750	−0.0643	0.0971	0.052*	
H4B	0.4424	0.0891	0.1094	0.052*	
C5	0.65262 (13)	0.06655 (12)	0.13208 (10)	0.0373 (2)	
C6	0.75128 (12)	0.01894 (12)	0.21271 (10)	0.0371 (2)	
C7	0.86725 (12)	0.08662 (13)	0.19491 (10)	0.0381 (2)	
H7	0.9692	0.0051	0.2097	0.046*	
C8	0.87933 (12)	0.17859 (13)	0.06498 (10)	0.0380 (2)	
C9	1.01106 (13)	0.22456 (14)	0.01719 (12)	0.0465 (3)	
C10	1.01073 (15)	0.32985 (16)	−0.10760 (13)	0.0549 (3)	
H10A	1.0736	0.2719	−0.1646	0.066*	
H10B	1.0605	0.3935	−0.1099	0.066*	
C11	0.85044 (15)	0.42923 (14)	−0.15076 (12)	0.0476 (3)	
C12	0.76661 (16)	0.32723 (14)	−0.13191 (11)	0.0467 (3)	
H12A	0.6587	0.3880	−0.1454	0.056*	
H12B	0.8143	0.2703	−0.1911	0.056*	
C13	0.77272 (13)	0.22295 (13)	−0.00826 (10)	0.0386 (2)	
C14	0.38365 (17)	0.05031 (19)	0.34291 (15)	0.0644 (4)	
H14A	0.4406	0.1052	0.3437	0.097*	
H14B	0.3532	0.0020	0.4246	0.097*	
H14C	0.2925	0.1174	0.3032	0.097*	
C15	0.39565 (18)	−0.15434 (18)	0.27371 (14)	0.0616 (4)	
H15A	0.3623	−0.1999	0.3555	0.092*	
H15B	0.4616	−0.2302	0.2333	0.092*	
H15C	0.3064	−0.0880	0.2316	0.092*	
C16	0.75801 (18)	0.54296 (16)	−0.07819 (15)	0.0622 (4)	
H16A	0.6554	0.6002	−0.1024	0.093*	
H16B	0.8098	0.6083	−0.0931	0.093*	
H16C	0.7506	0.4920	0.0066	0.093*	
C17	0.8681 (2)	0.51037 (19)	−0.28546 (14)	0.0716 (4)	
H17A	0.7671	0.5682	−0.3125	0.107*	
H17B	0.9287	0.4387	−0.3308	0.107*	
H17C	0.9196	0.5752	−0.2976	0.107*	
C18	0.82627 (12)	0.18056 (13)	0.28097 (10)	0.0375 (2)	
C19	0.92946 (14)	0.15185 (14)	0.35771 (11)	0.0431 (3)	
H19	1.0239	0.0699	0.3589	0.052*	0.932 (3)
C20	0.89720 (16)	0.24020 (16)	0.43221 (12)	0.0514 (3)	
H20	0.9683	0.2190	0.4825	0.062*	
C21	0.75792 (18)	0.35938 (16)	0.42986 (12)	0.0528 (3)	
C22	0.64974 (16)	0.39404 (15)	0.35770 (13)	0.0540 (3)	
H22	0.5549	0.4753	0.3580	0.065*	
C23	0.68774 (14)	0.30316 (14)	0.28486 (11)	0.0454 (3)	
H23	0.6157	0.3255	0.2350	0.054*	0.068 (3)

### Atomic displacement parameters (Å^2^)

**Table:**

	*U*^11^	*U*^22^	*U*^33^	*U*^12^	*U*^13^	*U*^23^
F1	0.0438 (5)	0.0656 (6)	0.0797 (7)	0.0015 (4)	−0.0322 (4)	−0.0315 (5)
F1'	0.058 (8)	0.079 (10)	0.093 (11)	−0.011 (7)	−0.035 (7)	−0.023 (8)
F2	0.1159 (8)	0.0833 (7)	0.0739 (6)	−0.0448 (6)	−0.0094 (6)	−0.0435 (5)
O1	0.0698 (6)	0.0637 (6)	0.0568 (6)	−0.0285 (5)	−0.0337 (5)	0.0026 (5)
O2	0.0467 (4)	0.0482 (5)	0.0423 (4)	−0.0277 (4)	−0.0157 (4)	−0.0010 (4)
O3	0.0419 (5)	0.0823 (7)	0.0837 (7)	−0.0312 (5)	−0.0171 (5)	−0.0095 (6)
C1	0.0434 (6)	0.0413 (6)	0.0406 (6)	−0.0136 (5)	−0.0113 (5)	−0.0101 (5)
C2	0.0513 (7)	0.0478 (7)	0.0417 (6)	−0.0244 (6)	−0.0080 (5)	−0.0031 (5)
C3	0.0430 (6)	0.0481 (7)	0.0434 (6)	−0.0237 (5)	−0.0058 (5)	−0.0090 (5)
C4	0.0473 (6)	0.0493 (7)	0.0423 (6)	−0.0274 (5)	−0.0126 (5)	−0.0076 (5)
C5	0.0407 (6)	0.0386 (6)	0.0369 (6)	−0.0189 (5)	−0.0086 (4)	−0.0081 (5)
C6	0.0359 (5)	0.0389 (6)	0.0401 (6)	−0.0151 (5)	−0.0085 (4)	−0.0116 (5)
C7	0.0308 (5)	0.0386 (6)	0.0460 (6)	−0.0105 (4)	−0.0119 (4)	−0.0121 (5)
C8	0.0329 (5)	0.0390 (6)	0.0450 (6)	−0.0139 (4)	−0.0050 (4)	−0.0149 (5)
C9	0.0343 (5)	0.0485 (7)	0.0593 (8)	−0.0164 (5)	−0.0029 (5)	−0.0197 (6)
C10	0.0447 (7)	0.0595 (8)	0.0625 (8)	−0.0281 (6)	0.0038 (6)	−0.0163 (7)
C11	0.0523 (7)	0.0446 (7)	0.0515 (7)	−0.0272 (6)	−0.0044 (6)	−0.0101 (6)
C12	0.0575 (7)	0.0482 (7)	0.0432 (7)	−0.0290 (6)	−0.0103 (5)	−0.0086 (5)
C13	0.0394 (6)	0.0396 (6)	0.0425 (6)	−0.0192 (5)	−0.0056 (5)	−0.0124 (5)
C14	0.0524 (8)	0.0734 (10)	0.0679 (9)	−0.0223 (7)	0.0030 (7)	−0.0303 (8)
C15	0.0641 (9)	0.0684 (9)	0.0636 (9)	−0.0449 (8)	−0.0104 (7)	−0.0041 (7)
C16	0.0664 (9)	0.0459 (7)	0.0798 (10)	−0.0208 (7)	−0.0154 (8)	−0.0208 (7)
C17	0.0896 (12)	0.0714 (10)	0.0602 (9)	−0.0517 (10)	−0.0078 (8)	0.0005 (8)
C18	0.0352 (5)	0.0392 (6)	0.0417 (6)	−0.0159 (5)	−0.0115 (4)	−0.0085 (5)
C19	0.0396 (6)	0.0474 (7)	0.0462 (6)	−0.0201 (5)	−0.0148 (5)	−0.0065 (5)
C20	0.0622 (8)	0.0639 (8)	0.0438 (7)	−0.0379 (7)	−0.0165 (6)	−0.0080 (6)
C21	0.0731 (9)	0.0549 (8)	0.0443 (7)	−0.0352 (7)	−0.0045 (6)	−0.0180 (6)
C22	0.0556 (8)	0.0455 (7)	0.0578 (8)	−0.0127 (6)	−0.0085 (6)	−0.0191 (6)
C23	0.0411 (6)	0.0476 (7)	0.0489 (7)	−0.0130 (5)	−0.0157 (5)	−0.0136 (5)

### Geometric parameters (Å, º)

**Table:**

F1—C23	1.3549 (13)	C11—C16	1.5239 (19)
F1'—C19	1.230 (14)	C11—C17	1.5287 (19)
F2—C21	1.3571 (15)	C11—C12	1.5330 (16)
O1—C1	1.2193 (14)	C12—C13	1.4843 (17)
O2—C5	1.3735 (13)	C12—H12A	0.9700
O2—C13	1.3736 (13)	C12—H12B	0.9700
O3—C9	1.2193 (15)	C14—H14A	0.9600
C1—C6	1.4638 (16)	C14—H14B	0.9600
C1—C2	1.5065 (17)	C14—H14C	0.9600
C2—C3	1.5332 (17)	C15—H15A	0.9600
C2—H2A	0.9700	C15—H15B	0.9600
C2—H2B	0.9700	C15—H15C	0.9600
C3—C14	1.522 (2)	C16—H16A	0.9600
C3—C15	1.5292 (17)	C16—H16B	0.9600
C3—C4	1.5299 (17)	C16—H16C	0.9600
C4—C5	1.4851 (15)	C17—H17A	0.9600
C4—H4A	0.9700	C17—H17B	0.9600
C4—H4B	0.9700	C17—H17C	0.9600
C5—C6	1.3389 (14)	C18—C23	1.3819 (17)
C6—C7	1.5112 (14)	C18—C19	1.3904 (14)
C7—C8	1.5089 (16)	C19—C20	1.3804 (18)
C7—C18	1.5207 (16)	C19—H19	0.9300
C7—H7	0.9800	C20—C21	1.363 (2)
C8—C13	1.3388 (15)	C20—H20	0.9300
C8—C9	1.4684 (15)	C21—C22	1.3685 (19)
C9—C10	1.5009 (19)	C22—C23	1.3730 (18)
C10—C11	1.5310 (19)	C22—H22	0.9300
C10—H10A	0.9700	C23—H23	0.9300
C10—H10B	0.9700		
			
C5—O2—C13	118.21 (8)	C11—C12—H12A	109.2
O1—C1—C6	120.42 (11)	C13—C12—H12B	109.2
O1—C1—C2	121.07 (11)	C11—C12—H12B	109.2
C6—C1—C2	118.44 (9)	H12A—C12—H12B	107.9
C1—C2—C3	114.85 (10)	C8—C13—O2	122.43 (10)
C1—C2—H2A	108.6	C8—C13—C12	125.78 (10)
C3—C2—H2A	108.6	O2—C13—C12	111.79 (9)
C1—C2—H2B	108.6	C3—C14—H14A	109.5
C3—C2—H2B	108.6	C3—C14—H14B	109.5
H2A—C2—H2B	107.5	H14A—C14—H14B	109.5
C14—C3—C15	109.58 (11)	C3—C14—H14C	109.5
C14—C3—C4	110.53 (11)	H14A—C14—H14C	109.5
C15—C3—C4	108.14 (10)	H14B—C14—H14C	109.5
C14—C3—C2	110.07 (11)	C3—C15—H15A	109.5
C15—C3—C2	110.10 (11)	C3—C15—H15B	109.5
C4—C3—C2	108.39 (10)	H15A—C15—H15B	109.5
C5—C4—C3	113.01 (9)	C3—C15—H15C	109.5
C5—C4—H4A	109.0	H15A—C15—H15C	109.5
C3—C4—H4A	109.0	H15B—C15—H15C	109.5
C5—C4—H4B	109.0	C11—C16—H16A	109.5
C3—C4—H4B	109.0	C11—C16—H16B	109.5
H4A—C4—H4B	107.8	H16A—C16—H16B	109.5
C6—C5—O2	123.04 (9)	C11—C16—H16C	109.5
C6—C5—C4	125.44 (10)	H16A—C16—H16C	109.5
O2—C5—C4	111.51 (9)	H16B—C16—H16C	109.5
C5—C6—C1	118.82 (10)	C11—C17—H17A	109.5
C5—C6—C7	122.11 (10)	C11—C17—H17B	109.5
C1—C6—C7	119.06 (9)	H17A—C17—H17B	109.5
C8—C7—C6	108.90 (9)	C11—C17—H17C	109.5
C8—C7—C18	110.47 (9)	H17A—C17—H17C	109.5
C6—C7—C18	113.06 (9)	H17B—C17—H17C	109.5
C8—C7—H7	108.1	C23—C18—C19	115.53 (11)
C6—C7—H7	108.1	C23—C18—C7	122.63 (9)
C18—C7—H7	108.1	C19—C18—C7	121.79 (10)
C13—C8—C9	118.38 (11)	F1'—C19—C20	119.4 (6)
C13—C8—C7	122.62 (10)	F1'—C19—C18	118.0 (7)
C9—C8—C7	118.98 (10)	C20—C19—C18	122.57 (12)
O3—C9—C8	120.69 (12)	C20—C19—H19	118.7
O3—C9—C10	121.52 (11)	C18—C19—H19	118.7
C8—C9—C10	117.76 (10)	C21—C20—C19	117.98 (11)
C9—C10—C11	115.71 (10)	C21—C20—H20	121.0
C9—C10—H10A	108.4	C19—C20—H20	121.0
C11—C10—H10A	108.4	F2—C21—C20	119.12 (12)
C9—C10—H10B	108.4	F2—C21—C22	118.00 (13)
C11—C10—H10B	108.4	C20—C21—C22	122.88 (12)
H10A—C10—H10B	107.4	C21—C22—C23	116.87 (12)
C16—C11—C17	109.55 (12)	C21—C22—H22	121.6
C16—C11—C10	110.27 (11)	C23—C22—H22	121.6
C17—C11—C10	110.20 (12)	F1—C23—C22	117.04 (11)
C16—C11—C12	110.34 (11)	F1—C23—C18	118.79 (11)
C17—C11—C12	109.15 (11)	C22—C23—C18	124.17 (11)
C10—C11—C12	107.29 (10)	C22—C23—H23	117.9
C13—C12—C11	112.16 (10)	C18—C23—H23	117.9
C13—C12—H12A	109.2		
			
O1—C1—C2—C3	155.46 (12)	C9—C10—C11—C16	68.74 (15)
C6—C1—C2—C3	−27.60 (16)	C9—C10—C11—C17	−170.19 (12)
C1—C2—C3—C14	−70.82 (14)	C9—C10—C11—C12	−51.47 (15)
C1—C2—C3—C15	168.27 (11)	C16—C11—C12—C13	−70.88 (14)
C1—C2—C3—C4	50.17 (14)	C17—C11—C12—C13	168.68 (12)
C14—C3—C4—C5	73.46 (13)	C10—C11—C12—C13	49.28 (14)
C15—C3—C4—C5	−166.59 (11)	C9—C8—C13—O2	174.81 (10)
C2—C3—C4—C5	−47.24 (14)	C7—C8—C13—O2	−6.63 (17)
C13—O2—C5—C6	9.76 (16)	C9—C8—C13—C12	−5.94 (18)
C13—O2—C5—C4	−168.91 (10)	C7—C8—C13—C12	172.62 (11)
C3—C4—C5—C6	23.38 (17)	C5—O2—C13—C8	−7.66 (16)
C3—C4—C5—O2	−157.99 (10)	C5—O2—C13—C12	173.00 (10)
O2—C5—C6—C1	−176.36 (10)	C11—C12—C13—C8	−23.44 (18)
C4—C5—C6—C1	2.12 (18)	C11—C12—C13—O2	155.88 (10)
O2—C5—C6—C7	2.50 (18)	C8—C7—C18—C23	63.24 (14)
C4—C5—C6—C7	−179.02 (11)	C6—C7—C18—C23	−59.07 (15)
O1—C1—C6—C5	176.86 (12)	C8—C7—C18—C19	−114.03 (11)
C2—C1—C6—C5	−0.10 (17)	C6—C7—C18—C19	123.66 (11)
O1—C1—C6—C7	−2.04 (17)	C23—C18—C19—F1'	−178.2 (9)
C2—C1—C6—C7	−179.00 (10)	C7—C18—C19—F1'	−0.8 (9)
C5—C6—C7—C8	−14.60 (15)	C23—C18—C19—C20	−0.60 (18)
C1—C6—C7—C8	164.25 (10)	C7—C18—C19—C20	176.85 (11)
C5—C6—C7—C18	108.58 (12)	F1'—C19—C20—C21	177.9 (9)
C1—C6—C7—C18	−72.56 (13)	C18—C19—C20—C21	0.34 (19)
C6—C7—C8—C13	16.67 (15)	C19—C20—C21—F2	179.57 (11)
C18—C7—C8—C13	−108.05 (12)	C19—C20—C21—C22	0.3 (2)
C6—C7—C8—C9	−164.77 (10)	F2—C21—C22—C23	−179.89 (12)
C18—C7—C8—C9	70.50 (12)	C20—C21—C22—C23	−0.6 (2)
C13—C8—C9—O3	−172.78 (12)	C21—C22—C23—F1	−179.17 (12)
C7—C8—C9—O3	8.60 (18)	C21—C22—C23—C18	0.3 (2)
C13—C8—C9—C10	5.27 (17)	C19—C18—C23—F1	179.75 (11)
C7—C8—C9—C10	−173.35 (11)	C7—C18—C23—F1	2.32 (18)
O3—C9—C10—C11	−156.79 (13)	C19—C18—C23—C22	0.26 (19)
C8—C9—C10—C11	25.18 (17)	C7—C18—C23—C22	−177.17 (12)

### Hydrogen-bond geometry (Å, º)

**Table:**

*D*—H···*A*	*D*—H	H···*A*	*D*···*A*	*D*—H···*A*
C20—H20···O1^i^	0.93	2.38	3.3075 (15)	177

Symmetry code: (i) −*x*+2, −*y*, −*z*+1.
